# Supplementary material for: Dynamical compensation in physiological circuits
Source: Mol Syst Biol. 2016 Nov 8;12(11):886. doi: 10.15252/msb.20167216 (PMC5147051; doi:10.15252/msb.20167216)
Supplement: Supplementary file 1 — Appendix [file MSB-12-886-s001.pdf]

Appendix “Dynamical compensation in biological circuits”

Omer Karin, Avital Swisa, Benjamin Glaser, Yuval Dor, Uri Alon

Table of Contents

Section 1. Proof of DC in a general class of models. 2

Section 2. Dynamical compensation in detailed model of the glucose-insulin system. 4

Section 3. Ultrasensitive drop in beta cell removal around G=5mM enables robustness of the feedback loop itself to variation in parameters 5

Section 4. Effects of variability in endogenous glucose production rate and insulin degradation rate on glucose dynamics. 7

Section 5. Effects of different insulin sensitivity for hepatic and muscle tissue on glucose dynamics. 8

Section 6. DC mechanism in calcium homeostasis. 9

Section 7. Slow feedback from trophic hormone to stimulating tissue can potentially provide DC. 11

References 12

Section 1. Proof of DC in a general class of models.

We consider a more general class of models for systems that show DC with respect to variation in the parameters *s,p*:

$$\dot{y} = f(u, y, sx) \quad [1]$$

$$\dot{x} = g(y, pZ, x) \quad [2]$$

$$\dot{Z} = Z \cdot h(y) \quad [3]$$

An input-response curve is the dynamics of $y(t)$ for an input $u(t)$, starting from a steady-state with $u(0)=0$. The dynamical compensation feature (DC) is defined as independence of the input-response curve from the physiological parameters $s>0$ and $p>0$.

Theorem 1. The following conditions are sufficient for the system to show DC:

i. For all p, s, the system is stable at $y = y^*$, there exists a unique solution $sx = x^*$ for $f(0, y^*, sx) = 0$ and there exists a unique solution $psZ = Z^*$ for $g(y^*, psZ, x^*) = 0$
ii. A factorization condition on the function g: $g(y, psZ, sx) = sg(y, pZ, x)$

Proof. The factorization condition (ii) is sufficient for the dynamics of the system to be independent of *s,p* under the transformation: $\hat{x} = sx, \hat{Z} = psZ$:

$$\dot{y} = f(u, y, \hat{x}) \quad [4]$$

$$\dot{\hat{x}} = sg(y, pZ, x) = g(y, \hat{Z}, \hat{x}) \quad [5]$$

$$\dot{\hat{Z}} = \hat{Z} \cdot h(y) \quad [6]$$

From (i) it follows that the steady state of $\hat{x}_{st} = sx_{st} = x^*$ is constant and that at steady state $\hat{Z}_{st} = psZ_{st}$ is constant:

$$0 = g(y^*, Z^*, x^*) = g(y_{st}, Z^*, \hat{x}_{st})$$

So: $\hat{Z}_{st} = Z^*$. Thus, the variables $y, \hat{x}, \hat{Z}$ have the same initial conditions before and after changes in *s,p* and Eq. [4],[5],[6] depend only on the scaled variables. Thus, following a change in *s,p* the dynamics of $y(t)$ in response to an input signal $u(t)$ will be identical after adaptation. The system shows dynamical compensation.

Dynamical compensation can be extended to more complex regulatory networks. Consider the following regulatory network, in which x passes through multiple compartments (such a regulatory network may be relevant, for instance, for hormones such as insulin, which passes through compartments such as the portal vein and the interstitial fluid):

$$\dot{y} = f(u, y, sx_1, sx_2, \dots, sx_n) \quad [7]$$

$$\dot{x}_1 = g(y, pZ) - \mu_1 x_1 \quad [8]$$

$$\dot{x}_i = \eta_{i-1} x_{i-1} - \mu_i x_i \qquad i \in \{2, \dots, n\} \quad [9]$$

$$\dot{Z} = Z \cdot h(y) \quad [10]$$

Here the μ_i coefficients describe the sum of the degradation rate γ_i and the transport rate η_i.

Theorem 2. The following conditions are sufficient for the system described by Eq. [7-10] to show DC:

i. For all p, s, the system is stable at $y = y^*$, there exists a unique solution $sx_1 = x^*$ for $f(0, y^*, sx_1, \frac{\eta_1}{\mu_2} sx_1, \dots, \frac{\eta_1 \cdots \eta_{n-1}}{\mu_2 \cdots \mu_n} sx_1) = 0$ and there exists a unique solution $psZ = Z^*$ for $g(y^*, psZ) - \mu_1 x^* = 0$
ii. A factorization condition on the function g: $g(y, psZ) = sg(y, pZ)$

Proof. The factorization condition (ii) is sufficient for the dynamics of the system to be independent of *s,p* under the transformation: $\hat{x}_i = sx_i, \hat{Z} = psZ$:

$$\dot{y} = f(u, y, \hat{x}_1, \dots, \hat{x}_n) \quad [11]$$

$$\dot{\hat{x}}_1 = sg(y, pZ) - s\mu_1 x_1 = g(y, \hat{Z}) - \mu_1 \hat{x}_1 \quad [12]$$

$$\dot{\hat{x}}_i = s\eta_{i-1} x_{i-1} - s\mu_i x_i = \eta_{i-1} \hat{x}_{i-1} - \mu_i \hat{x}_i \qquad i \in \{2, \dots, n\} \quad [13]$$

$$\dot{\hat{Z}} = \hat{Z} \cdot h(y) \quad [14]$$

At steady state $\hat{x}_i = sx_i = \frac{\eta_1 \cdots \eta_{i-1}}{\mu_2 \cdots \mu_i} sx_1$, and thus from (i) it follows that the steady state of $\hat{x}_{1_{st}} = sx_{1_{st}} = x^*$ is constant. Thus, the steady state for each $\hat{x}_i$ is also constant. At steady state also $\hat{Z}_{st} = psZ_{st}$ is constant:

$$0 = g(y^*, Z^*) - c_1 x^* = g(y_{st}, Z^*) - c_1 \hat{x}_{1_{st}}$$

So: $\hat{Z}_{st} = Z^*$. The variables $y, \hat{x}_1, \ldots, \hat{x}_n, \hat{Z}$ have the same initial conditions before and after changes in s,p and Eq. [11-14] depend only on the scaled variables. Thus, following a change in s,p the dynamics of $y(t)$ in response to an input signal $u(t)$ will be identical after adaptation and the system shows dynamical compensation.

Section 2. Dynamical compensation in detailed model of the glucose-insulin system.

Here we present the detailed meal simulation model by Dalla Man et al. (Dalla Man et al., 2007). This model includes Eq. [1], [3-5], [10-11], [13-19], [23-27] from (Dalla Man et al., 2007) and Eq. [8] from (Man et al., 2006). The steady state constraints for the model are reported in Eq. [2], [6-9], [12] and [20-22] from (Dalla Man et al., 2007). The parameters for the model are given in Table 1 in (Dalla Man et al., 2007). The model describes the dynamics of glucose in response to a meal, and incorporates the transit of glucose through the gastro-intestinal tract, the secretion of insulin from beta cells and the effect of insulin and glucose on the liver and on the muscle and adipose tissues.

The original model did not include beta cell functional mass dynamics. We thus added to it an equation for the dynamics of beta cell functional mass, which we denote here as B (since the parameter β already exists in the model). The equation is the same as Eq. [9] in the main text:

$$\dot{B} = B(\lambda_+(G) - \lambda_-(G)) = B \cdot h(G)$$

with the production and removal rates $\lambda_+(G), \lambda_-(G)$ the same as specified in the methods section, with adjusted units. Beta cell functional mass B determines the rate of insulin secretion. In the detailed model, this rate is set by two constants β, K that determine the responsivity of beta cells to glucose and glucose rate of change respectively. We thus replaced them with $B\beta, BK$, so Eq. (25),(26) in the model become, respectively:

$$S_{po}(t) = \begin{cases} Y(t) + BK \cdot \dot{G}(t) + S_b & for\ \dot{G}(t) > 0 \\ Y(t) + S_b & for\ \dot{G}(t) \leq 0 \end{cases}$$

$$\dot{Y}(t) = \begin{cases} -\alpha \cdot [Y(t) - B\beta \cdot (G(t) - h)] & if\ \ B\beta \cdot (G(t) - h) \geq -S_b \\ -\alpha \cdot Y(t) - \alpha \cdot S_b & if\ \ B\beta \cdot (G(t) - h) < -S_b \end{cases}$$

We then tested whether this model has DC by simulating its meal response dynamics before and after a change in insulin sensitivity (Fig. EV1). The change in insulin sensitivity was simulated by halving the constants $V_{mx}, k_{p3}, k_{p4}, m_5$. The constant V_{mx} is the rate of insulin-dependent glucose utilization in remote compartments; the constants k_{p3}, k_{p4} represent hepatic sensitivity to insulin and portal insulin (respectively) and m_5 represents the rate of hepatic insulin extraction. We assumed here that the change in these parameters is coordinated (an uncoordinated change will be discussed in Appendix Section 4).

We simulated this model with a 24-hour meal input (Fig. EV1A). After the change in insulin sensitivity, the model was simulated for several months until beta cell functional mass reached its new steady state. We then compared the 24-hour dynamics of glucose (Fig. EV1B), insulin (Fig. EV1C) and insulin relative to its baseline (Fig. EV1D) before and after the change insulin sensitivity. We observe that glucose dynamics are precisely the same, indicating that adding the slow loop on beta cell functional mass provides DC to changes in insulin sensitivity.

Section 3. Ultrasensitive drop in beta cell removal around G=5mM enables robustness of the feedback loop itself to variation in parameters

We propose that the desired glucose fixed point is maintained via a switch-like drop in beta cell removal rates around $G=G_0$. This ultra-sensitivity has been observed experimentally in rodent beta cells (Efanova et al., 1998). Efanova et al. incubated beta cells from ob/ob mice and Wistar rats for 40 hours in different glucose concentrations. After the incubation the percentage of dead cells was measured (Fig. EV2A), showing a drop in beta

cell death with an apparent Hill coefficient of around 7. This switch-like drop means that for a large range of production rates, the curves describing the rates of removal and production of beta cells cross at the sharp drop-point of removal, keeping the steady state close to $G=G_0$ (Fig. EV2B). Perturbing the production rate should not significantly perturb the homeostatic set-point, yet it does affect the time it takes for the slow feedback loop to reach compensation.

What is the molecular mechanism for the ultra-sensitive drop in beta cell removal? Mild ultra-sensitivity can be provided by the glucose-sensing enzyme glucokinase (GCK) which controls both insulin secretion and beta cell mass (Froguel et al., 1993; Glaser et al., 1998; Porat et al., 2011; Terauchi et al., 2007). Further ultra-sensitivity may be gained by the energy-sensing AMP-activated protein kinase (AMPK) which lies downstream of GCK, and whose prolonged phosphorylation is associated with beta cell death and dysfunction (Fu et al., 2013).

Prolonged phosphorylation of AMPK causes beta cell death and dysfunction in cell lines (Kefas et al., 2003a; da SILVA XAVIER et al., 2003; Van de Casteele et al., 2003; Zhang et al., 2009) and primary beta cells and islets (Cai et al., 2007, 2008; Kefas et al., 2003b; Leclerc et al., 2004; Targonsky et al., 2006). Over-expression of AMPK causes beta cell death and dysfunction in vivo (Richards et al., 2005) (see review by Fu et al(Fu et al., 2013)). AMPK is activated by an increase in the ratio of AMP:ATP, which varies approximately as the square of the ratio ADP:ATP (Hardie et al., 1999). AMPK activation by AMP:ATP is cooperative with an apparent Hill coefficient of about $n_1=2.5\pm0.5$ (Hardie et al., 1999) and half-maximal activation $K_1\approx0.13$ (Hardie et al., 1999). Thus:

$$AMPK^P \approx \rho \frac{1}{1+\left(\frac{K_1}{AMP:ATP}\right)^{n_1}} \approx \rho \frac{1}{1+K_1^{n_1}(ATP:ADP)^{2n_1}} \quad [1]$$

The rate-limiting step in the production of ATP in beta cells is the phosphorylation of glucose by glucokinase, which occurs with an apparent Hill coefficient of about $n_2=1.7$

(Matschinsky et al., 1998) and halfway point K2=8.4mM (Matschinsky et al., 1998). Thus, the ATP:ADP ratio in the cell as a function of glucose can be approximated by the equation:

$$ATP{:}ADP \approx \mu \frac{1}{1+\left(\frac{K_2}{G}\right)^{n_2}} \quad [2]$$

Combining [1],[2] we get:

$$AMPK^P \approx \rho \frac{1}{1+K_1^{n_1}\mu^{2n_1}\left(1+\left(\frac{K_2}{G}\right)^{n_2}\right)^{-2n_1}} \quad [3]$$

This equation implies that the activation of AMPK as a function of glucose is similar to a Hill function with a Hill coefficient of about n=1.7·5=8.5±1.7. The half maximal activation of AMPK is determined by the constants K_1,K_2 as well as by μ. The parameter μ determines the ATP:ADP level as a function glucose, which is coupled to glucose stimulated insulin secretion (Maechler et al., 2006). By approximating μ≈10 from maximal glucose stimulated ATP:ADP production (Nilsson et al., 1996) we get a half maximal activation of G≈5mM. Thus, the robustness of AMPK activation around G=5mM depends on the robustness of μ, the maximal ATP:ADP ratio in the cell. Indeed, in mouse islets incubated at different glucose concentrations, AMPK is activated in a switch like manner around 5mM glucose, with an apparent Hill coefficient of at least 6 (Fu et al., 2009).

In order to determine hill coefficients for AMPK phosphorylation (Fu et al., 2009) and beta cell death (Efanova et al., 1998) as a function of glucose, we fitted the Hill function: $\frac{1-B}{1+\left(\frac{G}{K}\right)^n} + B$, where G is glucose concnetration. We estimated the parameters B,K,n by least-square estimates using the nls function of R.

Section 4. Effects of variability in endogenous glucose production rate and insulin degradation rate on glucose dynamics.

While the glucose dynamics presented in Eq. [7-9] in the main text have DC with respect to changes in insulin sensitivity (S_i) and insulin secretion (p), they do not have DC with respect to changes in insulin degradation (γ) and endogenous glucose production (u_0). To show this, we compared the simulated glucose dynamics in response to the same meal intake by individuals with diminished insulin degradation ($\gamma/8$), diminished endogenous glucose production ($u_0/2$) and wild-type (Fig. EV3). For each case we simulated the dynamics after the system reached its new steady state. We observe that the dynamics of individuals with diminished insulin degradation or diminished endogenous glucose production are not the same as wild-type, and thus the system does not have DC with respect to these parameters.

Section 5. Effects of different insulin sensitivity for hepatic and muscle tissue on glucose dynamics.

Here we examine the effect of an uncoordinated change in hepatic versus muscle insulin sensitivity. To do so we model glucose dynamics using a minimal model which is based on the best-fit model presented by Dalla Man et al. (Man et al., 2008). The model includes the suppression of hepatic glucose production by insulin:

$$\dot{G} = u_0 + u(t) - S_H I - (C + S_M I) \cdot G \quad [1]$$

where S_H represents hepatic insulin sensitivity and S_M represents skeletal muscle insulin sensitivity. Because the equation for beta cell functional mass can only reach steady state at $G=G_0$, glucose steady state G_0 does not depend on S_H and S_M. Temporal dynamics are, however, a function of the ratio S_M/S_H. For a given ratio $S_M/S_H=\delta$, we can replace: $S_M \leftarrow \delta S_H$, and the general theorem in (Box 1) applies and DC is achieved but the shape of the input-response profile depends on the ratio of the two insulin sensitivities. The response to a given

glucose challenge will be lower in amplitude and more rapid the higher muscle sensitivity S_M is relative to hepatic sensitivity S_H.

This expectation matches clinical observations on the glucose response of individuals with mis-coordinated sensitivity in the two tissues: hepatic resistance and muscle sensitivity vs. muscle resistance and hepatic sensitivity to insulin (Abdul-Ghani et al., 2008) (Fig. EV4). In (Fig. EV4A) we present the results of an oral glucose tolerance test of such individuals as reported by Abdul Ghani et al. (Abdul-Ghani et al., 2008). Individuals with hepatic insulin resistance and normal muscle insulin sensitivity (in the model - high S_M/S_H) showed a lower and more rapid glucose response than individuals with muscle insulin resistance and normal hepatic insulin sensitivity (in the model - low S_M/S_H). These clinical observations are comparable to the glucose dynamics that are simulated using Eq. [1] (Fig. EV4B) where hepatic insulin resistance was simulated by setting $S_H=0$ and muscle insulin resistance was simulated by setting $S_M=0$.

Interestingly, in subjects with impaired glucose metabolism, impaired glucose tolerance (IGT) is associated with muscle insulin resistance while impaired fasting glucose (IFG) is associated with hepatic insulin resistance (Abdul-Ghani et al., 2006). This is predicted by the above analysis – among individuals with insufficient insulin secretion, muscle insulin resistance can cause impaired postprandial responses even when fasting glucose is within the normal range. On the other hand, individuals with hepatic insulin resistance and insufficient insulin secretion might have high fasting glucose and still be glucose tolerant.

Section 6. DC mechanism in calcium homeostasis.

Calcium is maintained in tight homeostasis in the blood, around $[Ca^{2+}] = 1.2mM$. This homeostasis is maintained by the parathyroid (PT) gland, which secretes parathyroid

hormone (PTH). The parathyroid gland secretes PTH in response to a drop in plasma calcium. The increase in PTH causes an increase in calcium production by the bones and an increase in calcium reabsorption in the kidneys, as well as increased production of activated vitamin D, which increases calcium absorption in the intestine. Calcium dynamics can be represented using the following equations (Fig. EV5):

$$\dot{[\mathrm{Ca}^{2+}]} = s \cdot [\mathrm{PTH}] - (u_0 + u(t)) \cdot [\mathrm{Ca}^{2+}] \qquad [1]$$

where $[\mathrm{Ca}^{2+}]$ is the plasma calcium concentration, $[\mathrm{PTH}]$ is the PTH concentration, $u_0 + u(t)$ is the calcium consumption rate and s represents the effectiveness of the parathyroid hormone in increasing calcium production. The parameter s depends on the response of the kidney and bone tissues to the parathyroid hormone. Secretion of PTH is a modeled by the equation:

$$\dot{[\mathrm{PTH}]} = pM \cdot \rho([\mathrm{Ca}^{2+}]) - \gamma[\mathrm{PTH}] \qquad [2]$$

where $\rho([\mathrm{Ca}^{2+}])$ is a monotonically decreasing function of calcium, γ is the PTH removal rate and p is the PTH secretion per PT cell.

As was the case with beta cell functional mass, we assume here that the functional mass of the parathyroid gland is chiefly controlled plasma calcium. This assumption is supported by several experimental studies. Low calcium diet causes a 10-fold increase in PT-cell proliferation in rodents (Naveh-Many et al., 1995), while direct activation of the calcium receptor inhibits PT-cell proliferation (Chen, 2004; Chin et al., 2000; Wada, 2003; Wada et al., 1997, 2000) and increases PT-cell apoptosis (Mizobuchi et al., 2007). Thus, parathyroid mass dynamics can be modeled as follows:

$$\dot{M} = M \cdot h([\mathrm{Ca}^{2+}]) \qquad [3]$$

where $h([\mathrm{Ca}^{2+}])$ is the net parathyroid growth rate.

This model has DC if M is stable at the desired set point of plasma calcium, that is, if $h([\mathrm{Ca}^{2+}]) = 0$ at $\mathrm{Ca}^{2+} \approx 1.2\mathrm{mM}$ with a negative local slope. If this is the case, DC is

achieved because Eq. [1-3] satisfy the sufficient conditions for DC. Note the differences from the glucose homeostasis model: the PTH hormone causes an increase in the regulated variable whereas insulin causes a decrease; the sensitivity parameter s appears in different terms: the removal term for glucose and the production term for calcium. Despite these differences, DC can be achieved by proper feedback of the regulated variable on the mass of the regulating tissue.

Section 7. Slow feedback from trophic hormone to stimulating tissue can potentially provide DC.

The pituitary trophic hormones Adrenocorticotropic hormone (ACTH) and Thyroid-Stimulating Hormone (TSH) stimulate respectively the secretion of glucocorticoids by the adrenal cortex and the secretion of thyroid hormones by the thyroid gland. They also cause respective changes in the mass dynamics of these glands (2011) which are potentially analogous to the changes in beta cell functional mass dynamics in response to blood glucose levels. Thus, the level of TSH is regulated by a slow and fast feedback from the thyroid gland and the level of ACTH is regulated by a slow and fast feedback from the adrenal cortex.

These circuits have various clinical implications. The pathophysiology of Thyroid Hormone Resistance Syndrome includes enlargement of the thyroid gland as well as higher thyroid hormone levels, while TSH levels are normal (Beck-Peccoz and Chatterjee, 1994). A similar pathophysiology in the ACTH-adrenal circuit occurs in Generalized Glucocorticoid Resistance (Charmandari et al., 2008). Inability to close the TSH-thyroid feedback loop (for instance, due to iodine deficiency) causes a significant enlargement of the thyroid, and the same occurs for the adrenal cortex when ACTH levels cannot be lowered (2011). On the other hand, large exogenous intake of the secreting hormone leads atrophy in the secreting gland (Selye, 1940). These features are consistent with a DC mechanism.

References

Abdul-Ghani, M.A., Tripathy, D., and DeFronzo, R.A. (2006). Contributions of -Cell Dysfunction and Insulin Resistance to the Pathogenesis of Impaired Glucose Tolerance and Impaired Fasting Glucose. Diabetes Care *29*, 1130–1139.

Abdul-Ghani, M.A., Matsuda, M., and DeFronzo, R.A. (2008). Strong association between insulin resistance in liver and skeletal muscle in non-diabetic subjects. Diabet. Med. J. Br. Diabet. Assoc. *25*, 1289–1294.

Beck-Peccoz, P., and Chatterjee, V.K. (1994). The variable clinical phenotype in thyroid hormone resistance syndrome. Thyroid Off. J. Am. Thyroid Assoc. *4*, 225–232.

Cai, Y., Martens, G.A., Hinke, S.A., Heimberg, H., Pipeleers, D., and Van de Casteele, M. (2007). Increased oxygen radical formation and mitochondrial dysfunction mediate beta cell apoptosis under conditions of AMP-activated protein kinase stimulation. Free Radic. Biol. Med. *42*, 64–78.

Cai, Y., Wang, Q., Ling, Z., Pipeleers, D., McDermott, P., Pende, M., Heimberg, H., and Van de Casteele, M. (2008). Akt activation protects pancreatic beta cells from AMPK-mediated death through stimulation of mTOR. Biochem. Pharmacol. *75*, 1981–1993.

Charmandari, E., Kino, T., Ichijo, T., and Chrousos, G.P. (2008). Generalized Glucocorticoid Resistance: Clinical Aspects, Molecular Mechanisms, and Implications of a Rare Genetic Disorder. J. Clin. Endocrinol. Metab. *93*, 1563–1572.

Chen, R.A. (2004). Role of the calcium-sensing receptor in parathyroid gland physiology. AJP Ren. Physiol. *286*, F1005–F1011.

Chin, J., Miller, S.C., Wada, M., Nagano, N., Nemeth, E.F., and Fox, J. (2000). Activation of the calcium receptor by a calcimimetic compound halts the progression of secondary hyperparathyroidism in uremic rats. J. Am. Soc. Nephrol. JASN *11*, 903–911.

Dalla Man, C., Rizza, R.A., and Cobelli, C. (2007). Meal simulation model of the glucose-insulin system. IEEE Trans. Biomed. Eng. *54*, 1740–1749.

Efanova, I.B., Zaitsev, S.V., Zhivotovsky, B., Köhler, M., Efendić, S., Orrenius, S., and Berggren, P.O. (1998). Glucose and tolbutamide induce apoptosis in pancreatic beta-cells. A process dependent on intracellular Ca2+ concentration. J. Biol. Chem. *273*, 33501–33507.

Froguel, P., Zouali, H., Vionnet, N., Velho, G., Vaxillaire, M., Sun, F., Lesage, S., Stoffel, M., Takeda, J., and Passa, P. (1993). Familial hyperglycemia due to mutations in glucokinase. Definition of a subtype of diabetes mellitus. N. Engl. J. Med. *328*, 697–702.

Fu, A., Ng, A.C.-H., Depatie, C., Wijesekara, N., He, Y., Wang, G.-S., Bardeesy, N., Scott, F.W., Touyz, R.M., Wheeler, M.B., et al. (2009). Loss of Lkb1 in adult beta cells increases beta cell mass and enhances glucose tolerance in mice. Cell Metab. *10*, 285–295.

Fu, A., Eberhard, C.E., and Screaton, R.A. (2013). Role of AMPK in pancreatic beta cell function. Mol. Cell. Endocrinol. *366*, 127–134.

Glaser, B., Kesavan, P., Heyman, M., Davis, E., Cuesta, A., Buchs, A., Stanley, C.A., Thornton, P.S., Permutt, M.A., Matschinsky, F.M., et al. (1998). Familial Hyperinsulinism Caused by an Activating Glucokinase Mutation. N. Engl. J. Med. *338*, 226–230.

Hardie, D.G., Salt, I.P., Hawley, S.A., and Davies, S.P. (1999). AMP-activated protein kinase: an ultrasensitive system for monitoring cellular energy charge. Biochem. J. *338 (Pt 3)*, 717–722.

Kefas, B.A., Cai, Y., Ling, Z., Heimberg, H., Hue, L., Pipeleers, D., and Van de Casteele, M. (2003a). AMP-activated protein kinase can induce apoptosis of insulin-producing MIN6 cells through stimulation of c-Jun-N-terminal kinase. J. Mol. Endocrinol. *30*, 151–161.

Kefas, B.A., Heimberg, H., Vaulont, S., Meisse, D., Hue, L., Pipeleers, D., and Van de Casteele, M. (2003b). AICA-riboside induces apoptosis of pancreatic beta cells through stimulation of AMP-activated protein kinase. Diabetologia *46*, 250–254.

Leclerc, I., Woltersdorf, W.W., da Silva Xavier, G., Rowe, R.L., Cross, S.E., Korbutt, G.S., Rajotte, R.V., Smith, R., and Rutter, G.A. (2004). Metformin, but not leptin, regulates AMP-activated protein kinase in pancreatic islets: impact on glucose-stimulated insulin secretion. Am. J. Physiol. Endocrinol. Metab. *286*, E1023–E1031.

Maechler, P., Carobbio, S., and Rubi, B. (2006). In beta-cells, mitochondria integrate and generate metabolic signals controlling insulin secretion. Int. J. Biochem. Cell Biol. *38*, 696–709.

Man, C.D., Camilleri, M., and Cobelli, C. (2006). A System Model of Oral Glucose Absorption: Validation on Gold Standard Data. IEEE Trans. Biomed. Eng. *53*, 2472–2478.

Man, C.D., Toffolo, G., Basu, R., Rizza, R.A., and Cobelli, C. (2008). Use of labeled oral minimal model to measure hepatic insulin sensitivity. AJP Endocrinol. Metab. *295*, E1152–E1159.

Matschinsky, F.M., Glaser, B., and Magnuson, M.A. (1998). Pancreatic beta-cell glucokinase: closing the gap between theoretical concepts and experimental realities. Diabetes *47*, 307–315.

Mizobuchi, M., Ogata, H., Hatamura, I., Saji, F., Koiwa, F., Kinugasa, E., Koshikawa, S., and Akizawa, T. (2007). Activation of calcium-sensing receptor accelerates apoptosis in hyperplastic parathyroid cells. Biochem. Biophys. Res. Commun. *362*, 11–16.

Naveh-Many, T., Rahamimov, R., Livni, N., and Silver, J. (1995). Parathyroid cell proliferation in normal and chronic renal failure rats. The effects of calcium, phosphate, and vitamin D. J. Clin. Invest. *96*, 1786–1793.

Nilsson, T., Schultz, V., Berggren, P.O., Corkey, B.E., and Tornheim, K. (1996). Temporal patterns of changes in ATP/ADP ratio, glucose 6-phosphate and cytoplasmic free Ca2+ in glucose-stimulated pancreatic beta-cells. Biochem. J. *314 (Pt 1)*, 91–94.

Porat, S., Weinberg-Corem, N., Tornovsky-Babaey, S., Schyr-Ben-Haroush, R., Hija, A., Stolovich-Rain, M., Dadon, D., Granot, Z., Ben-Hur, V., White, P., et al. (2011). Control of pancreatic β cell regeneration by glucose metabolism. Cell Metab. *13*, 440–449.

Richards, S.K., Parton, L.E., Leclerc, I., Rutter, G.A., and Smith, R.M. (2005). Over-expression of AMP-activated protein kinase impairs pancreatic {beta}-cell function in vivo. J. Endocrinol. *187*, 225–235.

Selye, H. (1940). COMPENSATORY ATROPHY OF THE ADRENALS. J. Am. Med. Assoc. *115*, 2246.

da SILVA XAVIER, G., Leclerc, I., Varadi, A., Tsuboi, T., Moule, S.K., and Rutter, G.A. (2003). Role for AMP-activated protein kinase in glucose-stimulated insulin secretion and preproinsulin gene expression. Biochem. J. *371*, 761–774.

Targonsky, E.D., Dai, F., Koshkin, V., Karaman, G.T., Gyulkhandanyan, A.V., Zhang, Y., Chan, C.B., and Wheeler, M.B. (2006). alpha-lipoic acid regulates AMP-activated protein kinase and inhibits insulin secretion from beta cells. Diabetologia *49*, 1587–1598.

Terauchi, Y., Takamoto, I., Kubota, N., Matsui, J., Suzuki, R., Komeda, K., Hara, A., Toyoda, Y., Miwa, I., Aizawa, S., et al. (2007). Glucokinase and IRS-2 are required for compensatory cell hyperplasia in response to high-fat diet-induced insulin resistance. J. Clin. Invest. *117*, 246–257.

Topp, B., Promislow, K., deVries, G., Miura, R.M., and Finegood, D.T. (2000). A model of beta-cell mass, insulin, and glucose kinetics: pathways to diabetes. J. Theor. Biol. *206*, 605–619.

Van de Casteele, M., Kefas, B.A., Cai, Y., Heimberg, H., Scott, D.K., Henquin, J.C., Pipeleers, D., and Jonas, J.C. (2003). Prolonged culture in low glucose induces apoptosis of rat pancreatic beta-cells through induction of c-myc. Biochem. Biophys. Res. Commun. *312*, 937–944.

Visentin, R., Dalla Man, C., Basu, R., Basu, A., Rizza, R.A., and Cobelli, C. (2015). Hepatic insulin sensitivity in healthy and prediabetic subjects: from a dual- to a single-tracer oral minimal model. Am. J. Physiol. - Endocrinol. Metab. *309*, E161–E167.

Wada, M. (2003). Control of parathyroid cell growth by calcimimetics. Nephrol. Dial. Transplant. *18*, 13iii – 17.

Wada, M., Furuya, Y., Sakiyama, J., Kobayashi, N., Miyata, S., Ishii, H., and Nagano, N. (1997). The calcimimetic compound NPS R-568 suppresses parathyroid cell proliferation in rats with renal insufficiency. Control of parathyroid cell growth via a calcium receptor. J. Clin. Invest. *100*, 2977–2983.

Wada, M., Nagano, N., Furuya, Y., Chin, J., Nemeth, E.F., and Fox, J. (2000). Calcimimetic NPS R-568 prevents parathyroid hyperplasia in rats with severe secondary hyperparathyroidism. Kidney Int. *57*, 50–58.

Zhang, X., Sun, N., Wang, L., Guo, H., Guan, Q., Cui, B., Tian, L., Gao, L., and Zhao, J. (2009). AMP-activated protein kinase and pancreatic/duodenal homeobox-1 involved in insulin secretion under high leucine exposure in rat insulinoma beta-cells. J. Cell. Mol. Med. *13*, 758–770.

(2011). Williams textbook of endocrinology (Philadelphia, Pa: Saunders Elsevier).
