# Supplementary material for: Dynamical compensation in physiological circuits
Source: Mol Syst Biol. 2016 Nov 8;12(11):886. doi: 10.15252/msb.20167216 (PMC5147051; doi:10.15252/msb.20167216)
Supplement: Supplementary file 2 — Expanded View Figures PDF [file MSB-12-886-s002.pdf]

Expanded View Figures

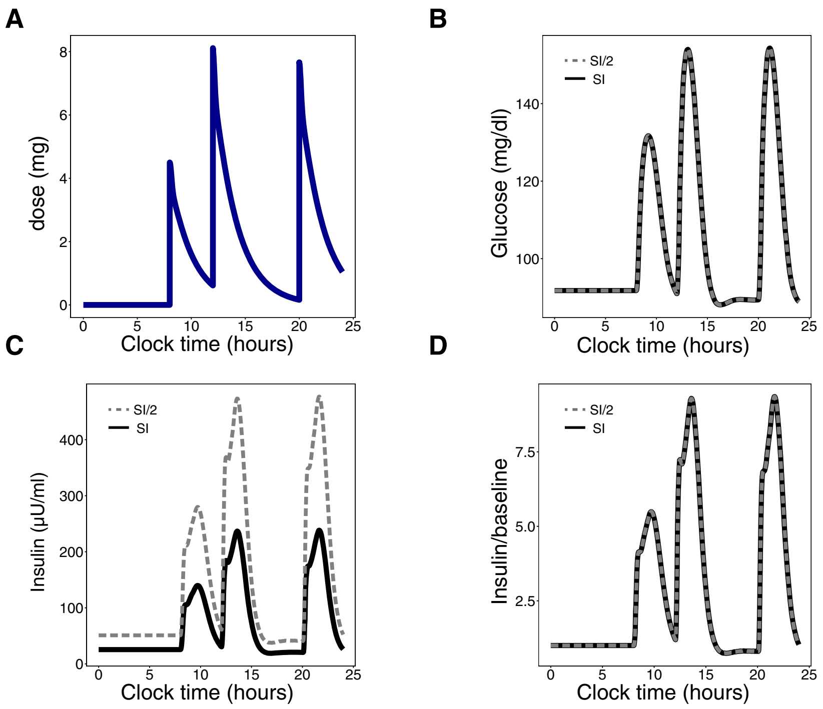

Figure EV1. Robustness of glucose temporal dynamics in a detailed meal simulation model of the glucose-insulin system.

A Input signal: Daily glucose rate of appearance.

B–D Dynamic profiles of plasma glucose concentration (B), plasma insulin concentration (C) and the fold of plasma insulin concentration relative to its baseline (D) in response to the input. Subjects are either normal (black) or insulin-resistant with beta-cell compensation (gray).

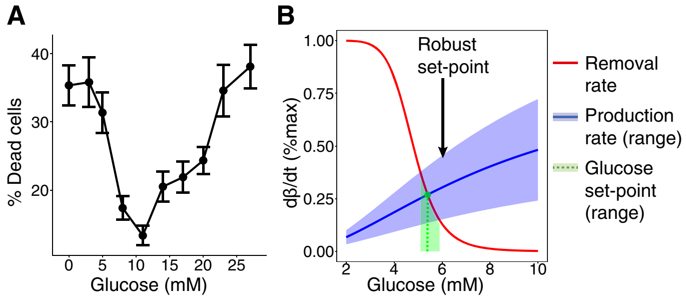

Figure EV2. Ultra-sensitive drop in beta-cell removal rate around the glucose concentration of 5 mM enables robustness to variation in beta-cell production rate.

A Percentage of dead cells after 40-h incubation of beta cells from ob/ob mice and Wistar rats in different glucose concentrations, from Efanova *et al* (1998). In each condition, a minimum of 1,000 cells from 3 different isolations was counted. Percentages of dead cells are expressed as mean ± SEM.

B The steady state of beta-cell functional mass (green) is at the intersection between beta-cell removal (red) and production (blue) rates. An ultra-sensitive change in beta-cell removal as a function of glucose concentration makes the steady-state glucose concentration robust to a large variation in beta-cell production—a 50% variation in beta-cell production (light blue area) corresponds to less than 10% variation in the homeostatic set point (light green area).

 © 2016 The Authors

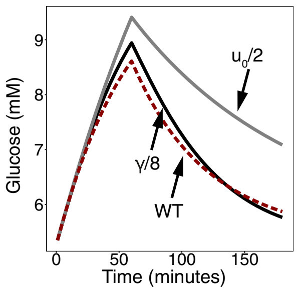

Figure EV3. The effect of a change in insulin degradation rate or endogenous glucose production rate on glucose dynamics.
Simulation of a glucose response to the same meal intake by a subject with reduced endogenous glucose production (gray), a subject with reduced insulin degradation (black), and a subject with normal parameters (red).

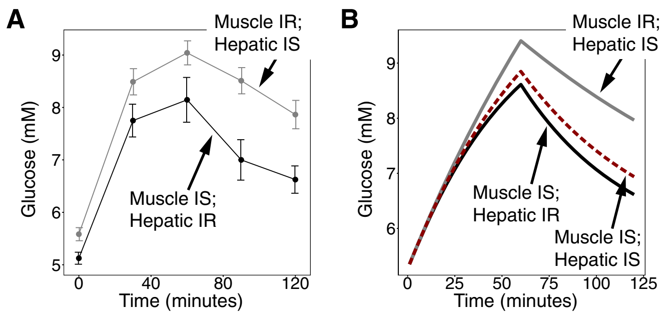

Figure EV4. The effect of hepatic insulin resistance relative to muscle insulin resistance on glucose dynamics.

A Plasma glucose concentration in an oral glucose tolerance test (OGTT) in 19 subjects with insulin resistance in skeletal muscle and normal hepatic insulin sensitivity (gray) and in 22 subjects with hepatic insulin resistance and normal insulin sensitivity in skeletal muscle (black). Figure from Abdul-Ghani *et al* (2008). Values are expressed as mean ± SEM.

B Simulation of glucose response from a subject with muscle insulin resistance (SM = 0) and normal hepatic insulin sensitivity (gray), a subject with hepatic insulin resistance (SH = 0) and normal muscle insulin sensitivity (black), and a subject with normal insulin sensitivity (red).

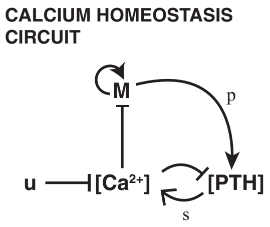

Figure EV5. Calcium homeostasis circuit.
Calcium ($[Ca^{2+}]$) suppresses the secretion of parathyroid hormone ([PTH]) as well as the proliferation of the parathyroid gland (M) which secretes the parathyroid hormone. Parathyroid hormone is secreted at a rate of *p* per cell and increases the production of calcium with effectiveness *s*. The circuit has an input *u* that represents calcium consumption.

© 2016 The Authors
